# Synthetic curcumin derivative DK1 possessed G2/M arrest and induced apoptosis through accumulation of intracellular ROS in MCF-7 breast cancer cells

**DOI:** 10.1186/s12935-017-0400-3

**Published:** 2017-02-21

**Authors:** Norlaily Mohd Ali, Swee Keong Yeap, Nadiah Abu, Kian Lam Lim, Huynh Ky, Ahmad Zaim Mat Pauzi, Wan Yong Ho, Sheau Wei Tan, Han Kiat Alan-Ong, Seema Zareen, Noorjahan Banu Alitheen, M. Nadeem Akhtar

**Affiliations:** 1 Faculty of Medicine and Health Sciences, Universiti Tunku Abdul Rahman, Lot PT 21144, Jalan Sungai Long, Bandar Sungai Long, Cheras, 43000 Kajang, Selangor Malaysia; 2 China-ASEAN College of Marine Sciences, Xiamen University Malaysia, Jalan Sunsuria, Bandar Sunsuria, 43900 Sepang, Selangor Malaysia; 3 UKM Molecular Biology Institute (UMBI), UKM Medical Centre, Jalan Yaa’cob Latiff, Bandar Tun Razak, Cheras, 56000 Kuala Lumpur, Malaysia; 4 Department of Agriculture Genetics and Breeding, College of Agriculture and Applied Biology, Cantho University, 3/2 Street, CanTho City, Vietnam; 5 Department of Cell and Molecular Biology, Faculty of Biotechnology and Biomolecular Sciences, University Putra Malaysia, 43400 Serdang, Selangor Malaysia; 6 School of Biomedical Sciences, The University of Nottingham Malaysia Campus, JalanBroga, 43500 Semenyih, Selangor Malaysia; 7 Institute of Bioscience, University Putra Malaysia, 43400 Serdang, Selangor Malaysia; 8 Faculty of Industrial Sciences & Technology, Universiti Malaysia Pahang, Lebuhraya Tun Razak, 26300 Kuantan Pahang, Malaysia

**Keywords:** DK1, ROS, Apoptosis, Cell cycle arrest, CDC2 phosphorylation

## Abstract

**Aims:**

Curcumin is a lead compound of the rhizomes of *Curcuma longa* and possess a broad range of pharmacological activities. Chemically, curcumin is 1,3-dicarbonyl class of compound, which exhibits keto-enol tautomerism. Despite of its strong biological properties, curcumin has yet been recommended as a therapeutic agent because of its poor bioavailability.

**Main methods:**

A curcumin derivative (*Z*)-3-hydroxy-1-(2-hydroxyphenyl)-3-phenylprop-2-en-1-one (DK1) was synthesized and its cytotoxicity was tested on breast cancer cell MCF-7 and normal cell MCF-10A using MTT assay. Meanwhile, cell cycle regulation and apoptosis on MCF-7 cell were evaluated using flow cytometry. Regulation of cell cycle and apoptosis related genes expression was investigated by quantitative real time polymerase chain reaction (qRT-PCR), western blot and caspases activity analyses. Activation of oxidative stress on MCF-7 were evaluated by measuring ROS and GSH levels.

**Key findings:**

DK1 was found to possess selective cytotoxicity on breast cancer MCF-7 cell than normal MCF-10A cell. Flow cytometry cell cycle and AnnexinV/PI analyses reported that DK1 effectively arrested MCF-7 at G2/M phase and induced apoptosis after 72 h of incubation than curcumin. Upregulation of p53, p21 and downregulation of PLK-1 subsequently promote phosphorylation of CDC2 which were found contributed to the arrest of G2/M phase. Moreover, increased of reactive oxygen species and reduced of antioxidant glutathione level correlate with apoptosis observed with raised of cytochrome c and active caspase 9.

**Significance:**

DK1 was found to be more effective in inducing cell cycle arrest and apoptosis against MCF-7 cell with much higher selectivity index of MCF-10A/MCF-7 than curcumin, which might be contributed by the overexpression of p53 protein.

## Background

Breast cancer contributes the highest to the total population of cancer cases and cancer-related mortality in women [1]. With the passage of time, it becomes highly aggressive disease and common cause of cancer death in women. The potential cause of breast cancer is believed due to the presence of estrogen receptor beta (ERb). Triple-negative breast cancer (TNBC) is a subtype of breast cancer defined by lack of expression of estrogen receptor alpha progesterone receptor and considered as the worst among all types of breast cancer [2–4]. Abnormal regulation of cell cycle and inhibition of apoptosis signaling pathways were commonly found in cancer cells. Chemotherapy targeting cancer cell with abnormal cell cycle profile or by inducing apoptosis have been widely used in cancer treatment [5]. However, the conventional chemo and hormone therapeutic agents have been reported associated with side effects, which were contributed by their cytotoxicity [6, 7]. Thus, effort to search for the alternative cytotoxic agents that target on the cell cycle progression and induce apoptosis specifically on cancer without harming normal cells is on-going [8].

Curcumin (diferuloylmethane), a member of the curcuminoid family, is the major active component of turmeric powder extracted from the rhizomes of *Curcuma longa.* It has been widely used as food additive and cosmetic ingredient. Over the past few decades, curcumin has been proven to be a remarkable drug for vast number of biological activities such as anti-carcinogenic [9–13], anti-malarial [14], antioxidant, anti-mutagenic, antibacterial [15, 16], anti-angiogenic [13], immunomodulatory [17] chemo-preventive [1, 12], anti- leishmaniasis [18] and anti-inflammatory effects [19].

Nevertheless, due to its partial solubility in water, curcumin has poor bioavailability and its clinical efficacy is rather limited [20]. Over the past few years, bioavailability issues related with poor absorption, distribution, metabolism and excretion of curcumin in serum levels and have limited its usage [21]. Although plants based natural compounds have been identified as potential source of anti-cancer agents due to its chemical diversity [22], chemically synthesized compounds have offered great potential to modify the natural compound structure to achieve better selectivity against cancer cell line [8]. Several curcumin derivatives were found to be more effective as anti-inflammatory agents than curcumin itself [19, 23]. Previously, we have reported the antihyperalgesic and antinociceptive activities of synthetic curcuminoid derivative, 2,6-bis-4-(hydroxy-3-methoxybenzilidine)-cyclohexanone in animal models [24, 25].

**Fig. 1:**
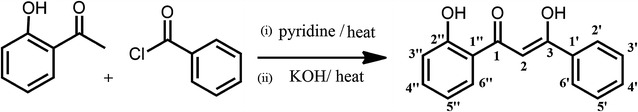
Synthesis of (*Z*)-3-hydroxy-1-(2-hydroxyphenyl)-3-phenylprop-2-en-1-one (DK1)

A simple curcuminoid, namely (*Z*)-3-hydroxy-1-(2-hydroxyphenyl)-3-phenylprop-2-en-1-one (DK1) was synthesized (Fig. 1). The compound DK1 was obtained as 100% pure crystals form and the structure was confirmed by single X-ray analysis [26]. Furthermore, the cytotoxicity and selectivity of DK1 on breast cancer and normal cell lines were also evaluated. Subsequently, the mechanism that alters cell cycle progression and apoptosis of DK1 treated MCF-7 breast cancer cell line was also determined. Our results provide the evidence that DK1 treatment induced p21 regulated G2/M phase arrest while promoted generation of reactive oxygen species (ROS) causing activation of DNA damage via p53 dependent apoptosis on MCF-7 breast cancer cell.

## Methods

### Synthesis and characterization of DK1

Compound DK1 was synthesized by Baker-Venkataraman rearrangement. A mixture of 2-hydroxyacetophenone 25.0 mol (3.5 g) and 21 mol (3.5 mL) of benzoyl chloride were added in a round bottle flask and stirred at 30 °C. About 30 mL pyridine anhydrous was added in a warm solution of above mixture and stirred for 1 h. After reaction completion, the product was neutralize with 5% HCl in 95 mL ice water and white crystalline compound was floating on the surface of water. The products 2-acetylphenyl benzoate was filter and washed with methanol and dried over sodium sulphate anhydrous. In the second step, the 2-acetylphenyl benzoate 12.5 mol (3.0 g) was dissolved in 15 mL pyridine anhydrous and stirred in a 100 mL beaker. About 0.5 g KOH was added in beaker during stirring condition and mixture were heated at 20 °C for 30 min. The products was neutralize with 15 mL acetic acid in ice water. The final the product was extracted with ethyl acetate and crystallized using methanol. The product was obtained as light yellow prism crystals and structure was confirmed by single X-ray and ^1^H-NMR data [26].

### Synthesis of (Z)-3-hydroxy-1-(2-hydroxyphenyl)-3-phenylprop-2-en-1-one (DK1)

Synthesized DK1 (Fig. 1) was the light yellow crystals with 96% yield and melting point ranging between 132 and 134 °C. IR (CHCl_3_)/cm: 36,500 (broad OH), 2955 (C–H stretch), 1658 (C=O), 1610 (C=C), 1516 (C=C), 1269 (C–O aromatic), 1074, 1001, ^1^H NMR (500 MHz, CDCl_3_): *δ* 15.50 (enol OH, C-3), 12.07 (OH, C-2″), 7.90 (s, ^1^H, C-2), 7.89 (d, 2H, *J* = 1.5 Hz, C-2′ & C-6′), 7.67 (m, 3H, C-3′, 4′, & C-5′), 7.77 (s, 1H, C-6″) *J* = 3.0 Hz, 1H, H-4), 6.98 (m, 3H C-3″, 4″, & C-5″). EIMS *m/z* (rel. int.) calcd for C_15_H_12_O_3_ [M^+^]: *m/z* = 240.2.

### Cell lines

Promyelocytic leukemia HL60, hepatoblastoma HepG2, breast cancer MCF-7 and MDA-MB-231 cells were purchased from ATCC (USA) and cultured in RPMI-1640 media (Sigma, USA), supplemented with 10% fetal bovine serum (FBS) (PAA, USA). Normal epithelial MCF-10A cells (ATCC, USA) was maintain in DMEM-F12 (Sigma, USA) supplemented with hydrocortisone (0.5 μg/mL), insulin (10 μg/mL), human epidermal growth factor (hEGF) (20 ng/mL) (Sigma, USA) and 10% FBS (PAA, USA).

### MTT cell viability assay and DK1 selective index

MTT cell viability assay [27] was used to evaluate the effect of DK1 on viability of HL-60, HepG2, MCF-7, MDA-MB-231 and MCF-10A cells. Briefly, each type of cells (8 × 10^4^cells/well) was seeded in 96-well plate in 37 °C CO_2_ incubator overnight. Then, DK1 was added at concentration ranging between 200 and 3.125 µM by twofold serial dilution. Untreated control was prepared simultaneously. After that, the cells were incubated for 24, 48 and 72 h at 37 °C in 5% CO_2_ incubator. After the incubation period, all well was added with 20 μL of MTT solution (5 mg/mL) and further incubated for 3 h. Subsequently, 170 μL of supernatant from each well was discarded, 100 μL of dimethyl sulfoxide (DMSO) was added to solubilize the purple formazan crystal and the absorbance was measured at a wavelength of 570 nm by Enzyme-linked immunosorbent assay (ELISA) plate reader (Bio-tek instruments, USA). All cell lines were assayed for three biological replicates each with triplicates. Percentage of cell viability was calculated using the following formula:

$$ {\text{Cell viability }}\left( \% \right) = \left( {{\text{OD sample}}/{\text{OD control}}} \right) \times 100\% $$

IC_50_ value (concentration of DK1 that reduce 50% of cell viability compared to control cell) was determined from the graph of cell viability (%) vs DK1 concentration. Subsequently, selective index, which indicating selectivity of DK1 against cancerous and normal breast cell lines, was calculated by:

$$\begin{aligned} &{\text{Selective index }}\left( {\text{SI}} \right) \\ &= \frac{{\left( {{\text{IC}}_{50} \;{\text{of DK}}1{\text{ on normal MCF-}}10{\text{A cell line}}} \right)}}{{{\text{IC}}_{50} \;{\text{on breast cancerous cell line }}\left( {{\text{HL}}60/{\text{HepG}}2/{\text{MCF-}}7/{\text{MDA-MB-}}231} \right)}} \end{aligned}$$

### MCF-7 cell treatment

MCF-7 cell, which was the most sensitive cell line to DK1, were seeded overnight in six well plate at 8 × 10^4^ cells/mL. After that, 25 µM of DK1 was added to the MCF-7. Untreated control and curcumin at 30 µM treatment were prepared simultaneously. After 24, 48 or 72 h of incubation control and treated MCF-7 cells were detached using TrypLE (Invitrogen, USA), washed with PBS (Sigma, USA) and subjected to the following assays. Curcumin treated cells were harvested at 72 h.

### Light and fluorescent microscopic observation

Prior to harvest the cell, morphology of control and DK1 treated MCF-7 was observed using light microscope. In addition, harvested control and treated MCF-7 cells were resuspended in 100 μL of Phosphate buffer saline (PBS), stained with 10 μg/mL of Acridine orange (AO) and propidium iodide (PI) and viewed under fluorescent microscope (Nikon, Japan).

### Flow cytometry AnnexinV-FITC/PI apoptosis analysis

Apoptosis of DK1 treated MCF-7 was compared with the control cell by Flow cytometry AnnexinV-FITC/PI apoptosis assay. Briefly, harvested cells were resuspended in 100 μL of 1× binding buffer and stained with 5 μL each of AnnexinV-FITC and propidiumiodide. After 15 min of incubation, the cells were added with 400 μL of 1× binding buffer and subjected to BD FACS Calibur flow cytometer analysis using BD Cell Quest Pro software (Becton–Dickinson, USA).

### Intracellular glutathione (GSH) and reactive oxygen species (ROS) detection

Harvested cell was subjected to two times of freeze and thaw in 100 μL of PBS. The lysed cell was then pelleted and the supernatant was subjected to GSH and ROS quantification using Glutathione assay kit (Sigma, USA) and OxiSelect ROS assay kit (Cell Biolabs, USA) according to manufacturers’ protocol.

For GSH quantification, 10 μL of cell lysate supernatant was added with 150 μL of working solution (1.5 mg/mL DTNB solution, 6 units/mL glutathione reductase and 1× assay buffer), incubated for 5 min and added with 50 μL of NADPH solution (0.16 mg/mL). The absorbance was read at a wavelength of 412 nm by ELISA plate reader (Bio-Tek instrument, USA) for every minute in duration of 5 min. For intracellular ROS quantification, supernatant was added with 10 μM DCFH-DA for 30 min at 37 °C. Then, the fluorescence intensity of DCFH-DA was measured using microplate fluorometer (Thermo Scientific, USA) with a 485/538 nm filter. Fold change of GSH and ROS was calculated by dividing absorbance or fluorescence intensity of DK1 treated MCF-7 with untreated control MCF-7.

### Active caspase 9, cytochrome c

The level of active caspase 9 and cytochrome c of the control and DK1 treated MCF-7 were quantified using CaspGLOW Red Active Caspase-9 staining kit (BioVision, USA) and human cytochrome c platinum ELISA (eBioscience Affymetrix, USA), respectively according to manufacturers’ protocol. Fold change of caspase 9 and cytochrome c was calculated by dividing fluorescence intensity or absorbance of DK1 treated MCF-7 with untreated control MCF-7.

### Western blot analysis

Total protein was extracted from harvested cell with Radioimmunoprecipitation assay (RIPA) buffer supplemented with phosphatase inhibitor cocktail (Roche, Canada) and the concentration was quantified by Bradford assay (Sigma, USA). Then, 100 μg of extracted protein was subjected to sodium dodecyl sulfate polyacrylamide gel electrophoresis (SDS-PAGE) (Bio-Rad, USA), transferred to nitrocellulose membrane, block with 0.5% skimmed milk overnight, washed with Tris-buffered saline tween (TBST) buffer and incubated with primary antibodies (anti-CDC2, anti-pCDC2 (Tyr15), anti-p53, and anti-β-actin at a dilution of 1:1000 (Abcam, USA) for 1 h. After that, membranes were washed, incubated with 1:5000 diluted goat anti-rabbit IgG H&L conjugated to Alkaline Phosphatase (Abcam, USA) and developed under chemiluminescence condition (Super Signal West Pico, Pierce, USA) using the ChemiDoc XRS (Bio-Rad, USA). Differential level of evaluated protein in control and DK1 treated MCF-7 was calculated based on the bands intensity analyzed using the Quantity One 1D Analysis software (Bio-rad, USA).

### Flow cytometry cell cycle analysis

Cell cycle progression of control and DK1 treated MCF-7 was analysed using BD FACS Calibur flow cytometer (Becton–Dickinson, USA). Briefly, harvested cells were added with 250 μL of trypsin buffer with 10 min incubation, followed by 200 μL of trypsin inhibitor with RNase buffer with 10 min incubation, and finally stained with 200 μL of propidium iodide (PI) from BD Cycletest Plus kit (Becton–Dickinson, USA). All stained cells were subjected to BD FACS Calibur flow cytometer analysis using BD Cell Quest Pro software (Becton–Dickinson, USA).

### Quantitative reverse transcription real time PCR assay

RNeasy mini plus kit (Qiagen, USA) was used to extract total RNAs from control and DK1 treated MCF-7 cells. The extracted RNAs were subjected to nano-drop spectrophotometer (Eppendorf, Germany) for purity and concentration evaluation and were converted to cDNA using iScriptcDNA synthesis kit (Bio-Rad, USA). Reverse and forward primers for target genes (p21, PLK-1, WEE-1) and housekeeping genes (β-actin, 18srRNA and GAPDH) were listed in Table 1. The expression level of target genes was quantified by quantitative real time polymerase chain reaction (qRT-PCR) using SYBR select master mix (Life Technologies, USA) on iQ-5 Real Time PCR machine (Bio-Rad, USA). Differential expression of target genes were normalized against three housekeeping genes between control and DK1 treated MCF-7 cell using iQ5 optical system software (Bio-Rad, USA) [28].

**Table 1:** The accession number and sequence of the primers used in the quantitative real-time PCR assay

Accession number	Gene	Sequence
NM_001101.3	ACTB	F: 5′-AGAGCTACGAGCTGCCTGAC-3′ R: 5′-AGCACTGTGTTGGCGTACAG-3′
NM_002046.4	GAPDH	F: 5-GGATTTGGTCGTATTGGGC-3 R: 5-TGGAAGATGGTGATGGGATT-3
HQ387008.1	18S rRNA	F: 5-GTAACCCGTTGAACCCCATT-3 R: 5-CCATCCAATCGGTAGTAGCG -3
NM_005030.3	PLK1	F: 5-CCTGCACCGAAACCGAGTTAT-3 R: 5-CCGTCATATTCGACTTTGGTTGC-3
NM_001143976.1	WEE-1	F: 5-GGGAATTTGATGTGCGACAG-3 R: 5-CTTCAAGCTCATAATCACTGGCT-3
NM_001220778.1	p21	F: 5-TGTCCGTCAGAACCCATGC-3 R: 5-AAAGTCGAAGTTCCATCGCTC-3

### Statistical analysis

All assays were carried out in three biological replicates and statistical significant among different time point of DK1 treatment to control cell were analyzed using one-way analysis of variance (ANOVA) by SPSS 15 software. Duncan’s multiple range tests was used for post hoc analysis and *p* value <0.05 compared to untreated control was regarded as significant.

## Results

### DK1 selectively induced cytotoxicity against MCF-7 breast cancer cells

MTT assay was used to evaluate the cytotoxicity of DK1 on promyelocytic leukemia HL60, hepatoblastoma HepG2, breast cancer MCF-7 and MDA-MB-231 cell lines. Normal breast epithelial MCF-10A cell line was used as normal control for calculation of selectivity index (SI) of DK1 on normal cell comparing to cancerous cell lines. Table 2 summarized the IC_50_ value and selective index of DK1 and curcumin on all the tested cell lines at 24, 48 and 72 h. DK1 has shown time dependent cytotoxicity against all the tested cell lines with the best cytotoxic effect on breast cancer cells particularly on MCF-7 at 72 h (25 μM) while lowest sensitivity against normal MCF-10A cell at 24 h where no IC_50_ value was recorded up to 208 μM. In terms of selectivity, DK1 showed better cytotoxicity on both cancerous cells than normal cell with the highest selective index of 4.17 in MCF-7/MCF-10A at 72 h. On the other hand, curcumin was recorded with greater cytotoxic effect on all the tested cancer cell lines except MCF-7 cells compared to DK1. DK1, which was more effective in MCF-7 cells, possessed much higher selectivity index of MCF-10A/MCF-7 compared to curcumin. Since DK1 possessed the highest efficacy and selectivity against MCF-7 cell better than curcumin, details of cell cycle regulation and cell death induction of DK1 on MCF-7 were further evaluated at IC_50_ value of 25 µM at 24, 48 and 72 h.

**Table 2:** The values of IC_50_ of DK1 in MCF-7, MDA-MB231 and MCF-10A

Cell lines	24 h		48 h		72 h	
	DK1 (µM)	Curcumin (µM)	DK1 (µM)	Curcumin (µM)	DK1 (µM)	Curcumin (µM)
HL-60	137.61 ± 2.16	57.01 ± 3.75	123.85 ± 3.27	29.86 ± 3.11	73.39 ± 2.65	21.72 ± 1.76
HepG2	>150 ± 5.85	67.86 ± 2.88	137.61 ± 4.13	40.72 ± 2.76	64.22 ± 3.12	24.43 ± 2.25
MCF-7	96.83 ± 4.87	40.72 ± 3.24	33.33 ± 3.50	36.58 ± 2.31	25.00 ± 3.71	30.15 ± 2.36
MDA-MB-231	104.17 ± 5.23	35.29 ± 4.16	45.83 ± 4.66	21.72 ± 1.87	37.50 ± 4.82	21.72 ± 3.18
MCF-10A	>208	190.02 ± 3.67	125.83 ± 3.67	114.01 ± 3.57	104.17 ± 5.21	100.44 ± 3.17
Selective index of MCF-10A/MCF-7	>2.17	4.67	3.75	3.50	4.17	3.33
Selective index of MCF-10A/MDA	>2.00	5.38	2.72	5.25	2.77	4.63

### DK1 induced p53 mediated apoptosis through induction of ROS and inhibition of GSH

**Fig. 2:**
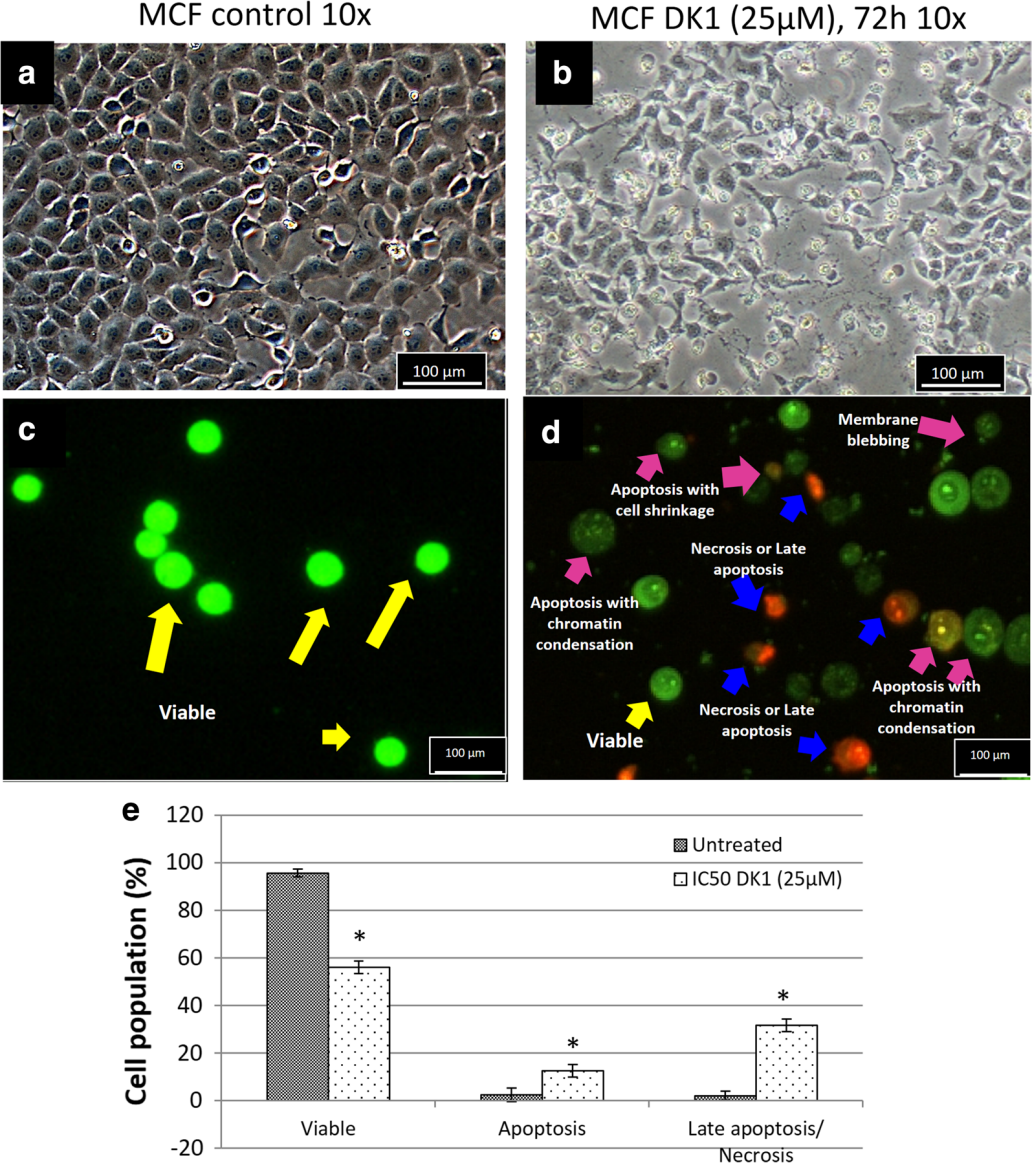
**a**, **b** Light and **c**, **d** fluorescent microscopic analysis of control and DK1 treated MCF-7 cell after 72 h of incubation. Cells in **c**, **d** were stained with acridine orange and propidium iodide. **e** *Bar chart* analysis of the percentage of viable, apoptotic and late apoptotic/necrotic of control and DK1 treated MCF-7 cells via fluorescent microscopic count of 200 cells. The experiment was done in triplicate and the data are expressed as mean ± SE with (*p < 0.05)

**Fig. 3:**
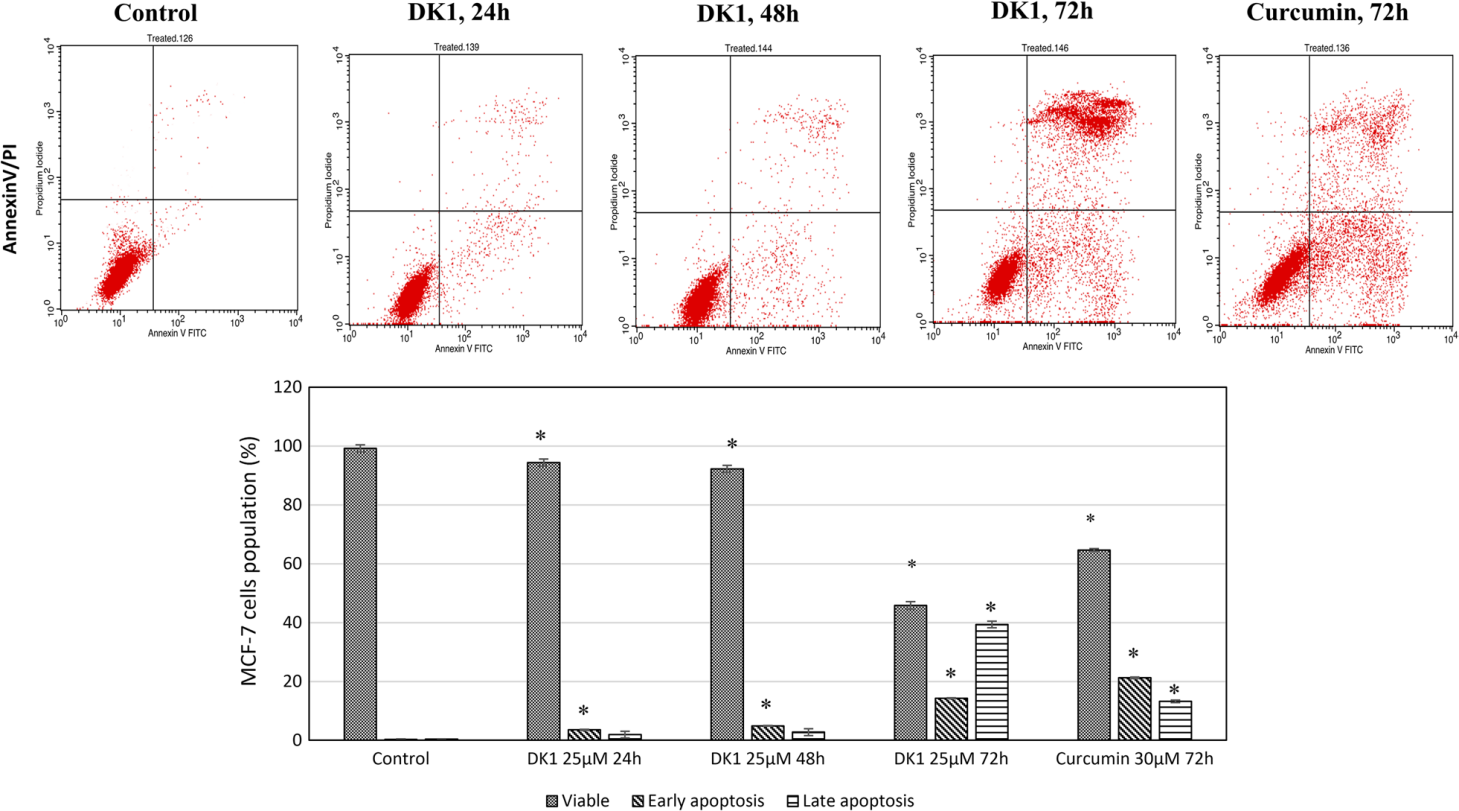
Flow cytometry Annexin V apoptosis of control and DK1 (25 µM) treated MCF-7. The experiment was done in triplicate and the data are expressed as mean ± SE with (*p < 0.05)

Light microscope observation presented a distinct different between morphology of control and 72 h-DK1 treated MCF-7 where control cell was confluent with well spread, adhered and extended morphology (Fig. 2a). In contrast, DK1 reduced the cell number and induced cell shrinkage on MCF-7 after 72 h of incubation (Fig. 2b). Fluorescence microscopic analysis using acridine orange and propidium iodide (PI) staining was used to evaluate the mode of cell death on MCF-7 induced by DK1. Acridine orange is a membrane permeable DNA dye that stained the viable cell as green. On the other hand, propidium iodide is a membrane impermeable DNA dye. It enters and binds with DNA to show red–orange colour when the cell loss the membrane and become permeable during apoptosis or necrosis [29]. Figure 2c shows that control MCF-7 cell was stained as green intact cell while DK1 treatment has induced apoptotic related morphological changes such as membrane blebbing, chromatin condensation and cell shrinkage (Fig. 2d). Random scoring based on 200 cells has recorded ~12.5 and ~31% of the cells were undergone apoptosis or late apoptosis/necrosis (Fig. 2e). This result was further supported by the flow cytometry AnnexinV/PI apoptosis assay through evaluation on the externalization of phosphatidylserine and loss of membrane integrity. Early apoptosis is indicated by binding of AnnexinV to externalise phosphatidylserine while late apoptosis or necrosis is shown by both binding of AnnexinV to phosphatidylserine and staining of propidium iodide to the DNA via loss of membrane integrity. Significant increase (p < 0.05) of early (~14%) and late apoptosis (~39%) was only observed after 72 h of DK1 treatment (Fig. 3). This result was similar to the percentage of apoptotic and late apoptotic cell as detected in fluorescent microscopic observation (Fig. 2e). Comparatively, 72 h of curcumin treatment only induced 15% of MCF-7 cells to late apoptosis, which was 2.6-folds lower than DK1 treatment at 72 h (Fig. 3).

**Fig. 4:**
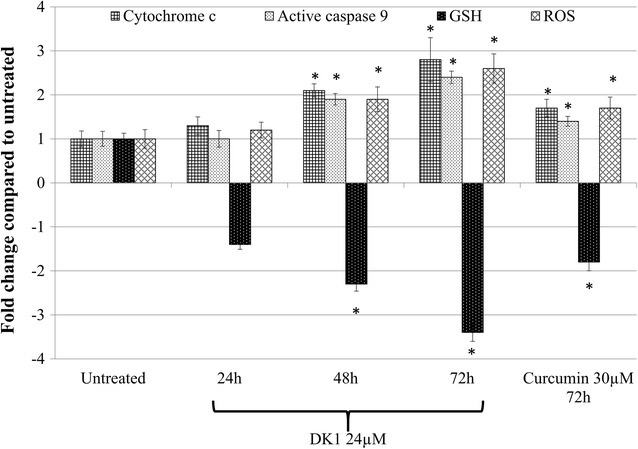
Detection of the activation of caspase 9, cytochrome c, GSH and ROS levels in the control and DK1 (25 μM) treated MCF-7 cells. The experiment was done in triplicate and the data are expressed as mean ± SE with (*p < 0.05)

**Fig. 5:**
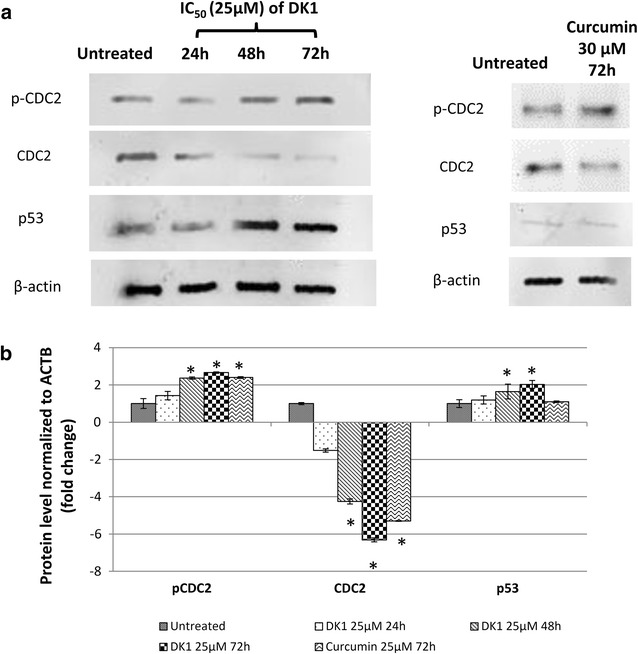
Differential protein expression of. **a** Western blot analysis of CDC2, p-CDC2 and p53 in MCF-7 treated with DK1 (25 μM) for 24, 48 and 72 h. **b** Differential protein level of control and DK1 (25 μM) treated MCF-7 cells normalised to β-actin. The experiment was done in triplicate and the data are expressed as mean ± SE with *p < 0.05

To determine the contribution of oxidative stress in the induction of apoptosis by DK1, level of ROS and antioxidant peptide GSH were determined. DK1 was able to significantly reduce the level of antioxidant peptide GSH (48 h: ~2.2-fold; 72 h: ~3.3-fold) and promote generation of ROS (48 h: ~1.9-fold; 72 h: ~2.6-fold) in the MCF-7 cell compared to control (Fig. 4). This effect was associated with promotion of p53 (48 h: ~1.6-fold; 72 h: ~2.0-fold) (Fig. 5), cytochrome c (48 h: ~2.1-fold; 72 h: ~2.8-fold) and active caspase 9 (48 h: ~1.9-fold; 72 h: ~2.4-fold) (Fig. 4) as observed in western blot, ELISA and fluorometry analyses, respectively. On the other hand, curcumin treatment induced a lower degree of deregulation of apoptosis related genes or proteins, particularly on the p53 protein compared to DK1 (Figs. 4, 5).

### DK1 induced G2/M cell cycle arrest through upregulation of p21 and downregulation of serine/threonine-protein kinase 1 (PLK1)

**Fig. 6:**
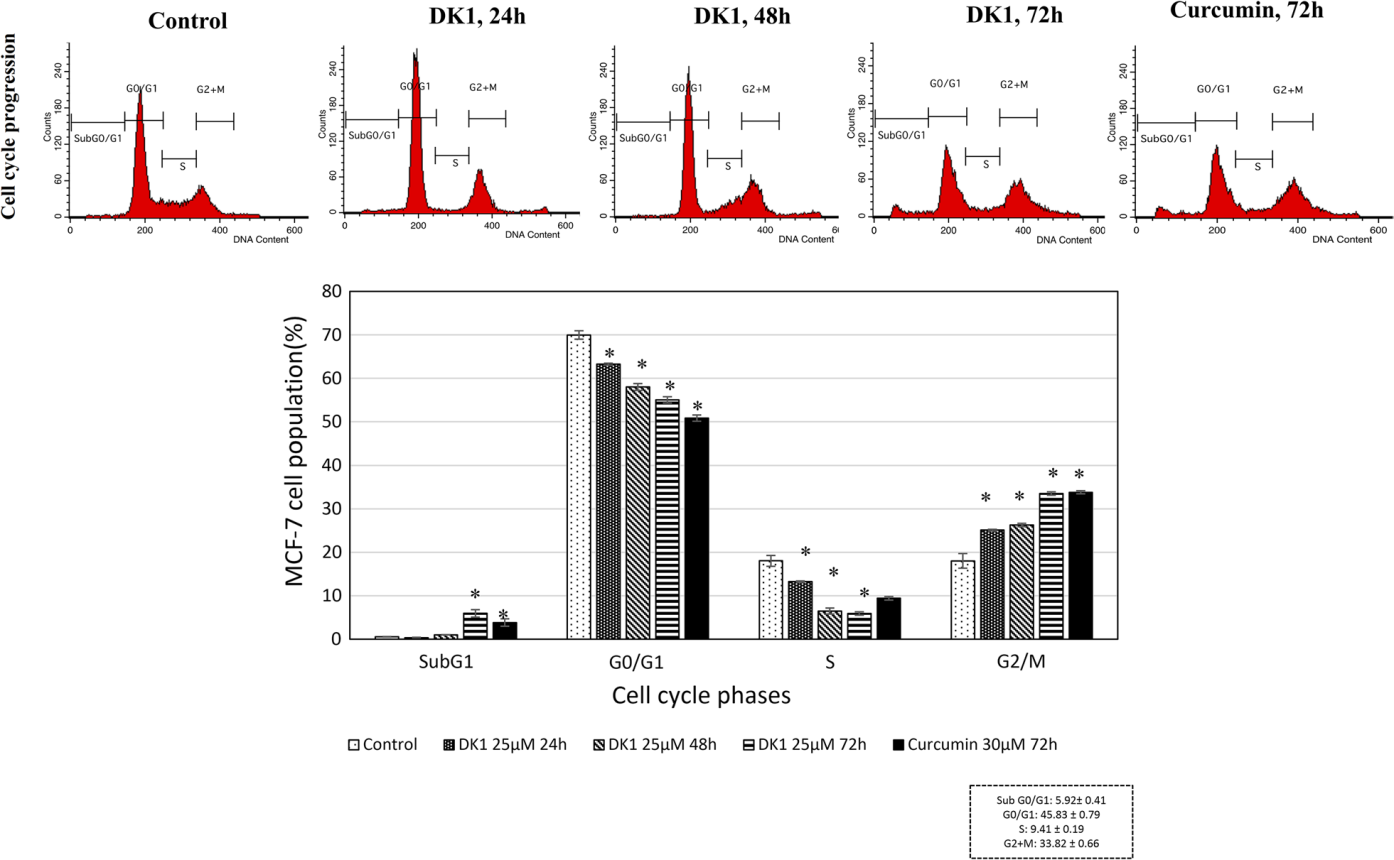
Flow cytometry cycle progression analyses of control and DK1 (25 µM) treated MCF-7. The experiment was done in triplicate and the data are expressed as mean ± SE with (*p < 0.05)

Figure 6 depicts representative cell cycle histograms of control and 25 µM DK1 treated MCF-7 cells following 24, 48 and 72 h incubation. DK1 treatment has recorded an increased in percentage of cell populations in G2/M phase (24 h: ~25%; 48 h: ~26%; 72 h: ~31%) accompanied by a reduction in G0/G1 phase compared to control cell (G2/M phase: ~18%). 72 h of curcumin treatment induced similar level of cell cycle arrest as DK1 treatment.

**Fig. 7:**
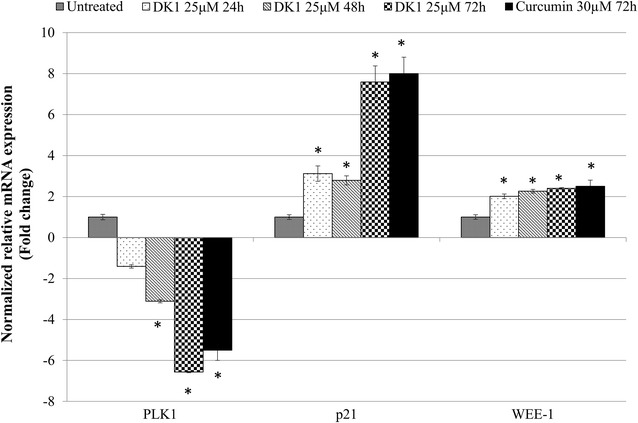
qPCR analysis of cell cycle related genes; PLK1, p21 and WEE-1 in MCF-7 treated with DK1 (25 μM) for 24, 48, and 72 h. The experiment was done in triplicate and the data are expressed as mean ± SE with (*p < 0.05)

The cell cycle analysis indicates the inhibitory effect of DK1 on proliferation of MCF-7 cells correlated with G2/M arrest. Thus, qRT-PCR and Western blot analysis were carried out to evaluate the role of cell cycle regulators associated with the DK1 induced G2/M arrest on MCF-7. qRT-PCR analysis shows that DK1 downregulated expression of PLK1 while enhanced the expression of p21 and WEE-1 significantly (p < 0.05) compared to control (Fig. 7). Furthermore, western blot analysis showed inhibition of CDC2 due to the phosphorylation on Tyr15 indicated by the reduction of CDC2 and the accumulation of phospho-CDC2 (Tyr15) after treating with DK1 for 48 and 72 h (Fig. 5). On the other hand, curcumin treatment induced a lower degree of deregulation of cell cycle regulation related genes compared to DK1 (Figs. 4, 7).

## Discussion

DK1, an analogue of curcumin with lower molecular weight was synthesized in this study andwas found most sensitive to MCF-7 cell comparing to MDA-MB-231 cell and normal MCF-10A cell. SI based on the IC_50_ value of the compound in normal and cancerous cell line demonstrates different efficacy of an evaluated compound in different type of cells. The higher SI value indicates better selectivity while low SI (generally <2) indicates the possibility of the compound to cause a toxicity side effect [8] and thus hold the value for further evaluation including preclinical studies using animal models. Even antiestrogen drug like 4-hydroxy tamoxifen has been reported with SI <2 [8] and this might be the reason that contributes to the side effect of this drug as recorded in clinical [6]. Thus, evaluating the SI value of a novel compound shall provide a good indication to further evaluate its antitumor effect both in vitro and in vivo. In this study, DK1 was recorded with SI values higher than 2 in both MCF-7 and MDA-MB-231 cell lines. Highest value of 4.17 SI was recorded in MCF-7 after 72 h of DK1 treatment. The SI value of DK1 on MCF-7 is higher than the other class of compound such as anthraquinone BHAQ (SI: 2.31) [30] and flavokawain A (SI: 3.78) [31]. Comparing to curcumin, DK1 showed better cytotoxicity as well as selectivity on MCF-7 cells. Previous study by Jia et al. [32] has reported that MCF-7 was resistant to curcumin, which is similar with our results. This result suggests that DK1 was a potential specific cytotoxic agent to MCF-7 cell line compared to curcumin. Thus, further studies to evaluate the details mode of cell death and mechanism involves were carried out.

Irregular cell cycle profile and development of anti-apoptosis have been commonly observed in cancer cell. Thus, capability to induce cell cycle arrest and promote apoptosis is the common criteria of potential chemotherapeutic agents [33]. Therefore, this study was focused to evaluate the regulation of cell cycle profile and induction of apoptosis by DK1 on MCF-7 cell. Microscopic and flow cytometry analyses have shown that 25 µM of DK1 induced cell death through apoptosis especially after 72 h of incubation. Accumulation of ROS and depletion of antioxidant peptide (Fig. 7) was observed in DK1 treated MCF-7 cell. A previous study has shown that excessive ROS induced by exogenous agents is the critical upstream event that leads to DNA damage and apoptosis of cancer cells through multiple signaling pathways [33].Tumor suppressor protein p53 has been identified as one of the downstream signaling protein activated by ROS accumulation on prostate cancer cell induced by a plant flavone, apigenin. This effect has given an idea that p53 is the predominant transcriptional regulator, which response to ROS-induced DNA damage [34]. Subsequently, p53 induces apoptosis by caspase 9 activation through the release of mitochondrial cytochrome c. Activation of caspase 9 in turn, leads to activation of caspase 3, degradation of many intracellular proteins, resulting in the morphological and biochemical changes of apoptosis [35]. This mechanism was observed in DK1 treated MCF-7 where significant (p < 0.05) increase of p53, cytochrome c, active caspase 9 were observed after 48 and 72 h of incubation. Subsequently, apoptosis related characteristics such as membrane blebbing, chromatin condensation, cell shrinkage and phosphatidylserine externalization were observed in MCF-7 after 72 h of DK1 treatment.

Other than apoptosis induction, DK1 was also found to arrest the cell cycle of MCF-7 at G2/M phase. DNA damage activates the G2 checkpoint mechanism to prevent cell cycle progression via p53 dependent mechanisms [36]. Cyclin kinase inhibitor, p21 is one of the downstream targets of tumor suppressor p53. Binding of CDC2 to cyclin B1 is the key checkpoint regulating progression from G2 to M transition while CDC-2 Tyr-15 phosphorylation leads to a G2 arrest [37]. Overexpression of p21 gene promote CDC2 (Tyr15) phosphorylation and thus activated G2/M cell cycle arrest [38]. Polo-like kinase 1 (PLK1) is a Ser/Thr kinase that plays pivotal roles in the activation of cyclinB1/CDC2 complex for transition of G2 to mitosis in cell cycle progression [39]. Overexpression of PLK1 has been reported in various types of cancers and thus it has been proposed as the potential target for cancer therapy. In addition, WEE-1 kinase also inactivates CDC2 activity to promote G2 arrest [37]. Taken together, the data from this study suggest that activation of p53 subsequently prevented cell cycle progression of MCF-7 at G2/M phase with the involvement of upregulation of p21 and WEE-1 gene together with inhibition of mitotic PLK1 followed by Tyr15 phosphorylation in CDC2.

Interestingly, previous study by Jia et al. [32] has reported that p21 and p27 induced PI3k/Akt signaling pathway play the important role in regulating cell cycle arrest and apoptosis induction in breast cancer cells. The expression level of p21 and other markers under PI3k/Akt signaling pathway were found lower in curcumin treated MCF-7 cells compared to curcumin treated MDA-MB-231 cells [32]. In this study, all the apoptosis and cell cycle related genes were found deregulated at a lower degree by curcumin compared to DK1, which might directly contributed to the better sensitivity of DK1 on MCF-7. A greater effect of DK1 in activating those genes may be directly contributed by the significant upregulation of p53, which was found absent in the curcumin treated MCF-7.

## Conclusions

In this study, DK1 was found to be more selective than curcumin in targeting MCF-7 cell by induction of apoptosis and G2/M cell cycle arrest, which might be contributed by the effective overexpression of p53 tumor suppressor protein. However, further study to evaluate the detail mechanisms and reproducibility of this effect in in vivo study is needed to establish a potential for clinical development.

## Abbreviations

ANOVA: one-way analysis of variant
AO: acridine orange
CDC2: cyclin-dependent kinase 1
CO2: carbon dioxide
DK1: (*Z*)-3-hydroxy-1-(2-hydroxyphenyl)-3-phenylprop-2-en-1-one
DMSO: dimethyl sulfoxide
ELISA: enzyme-linked immunosorbent assay
ERb: estrogen receptor beta
FBS: fetal bovine serum
GSH: glutathione
hEGF: human epidermal growth factor
IC_50_: concentration of DK1 that reduce 50% of cell viability compared to control cell
PBS: phosphate buffer saline
PCR: polymerase chain reaction
PI: propidium iodide
PLK1: serine/threonine-protein kinase 1
RIPA: radioimmunoprecipitation assay
ROS: reactive oxygen species
SDS-PAGE: sodium dodecyl sulfate polyacrylamide gel electrophoresis
SI: selective index
TBST: Tris-buffered saline tween
TNBC: triple-negative breast cancer
